# Styloid Process of the Temporal Bone: Morphometric Analysis and Clinical Implications

**DOI:** 10.1155/2016/8792725

**Published:** 2016-09-15

**Authors:** Antonio Luis Neto Custodio, Micena Roberta Miranda Alves e Silva, Mauro Henrique Abreu, Lucas Rodarte Abreu Araújo, Leandro Junqueira de Oliveira

**Affiliations:** ^1^Department of Morphology, Institute Of Biological Sciences, University Federal of Minas Gerais, Belo Horizonte, MG, Brazil; ^2^Department of Community and Preventive Dentistry, University Federal of Minas Gerais, Belo Horizonte, MG, Brazil; ^3^Postgraduate Program of Pontifical Catholic University of Minas Gerais, Belo Horizonte, MG, Brazil

## Abstract

*Objective*. To evaluate measures of the styloid process (SP) in Brazilian dry skulls.* Methods.* This study involves measurements of two points (lateral end posterior views) of 15 dry skulls held by the Morphology Department, Institute of Biological Sciences of Federal University of Minas Gerais.* Results.* There was a large variability for the length of left and right sides (in lateral and posterior views) of the styloid process. From the lateral view of the left and right styloid, the length of the SP ranged, respectively, from 10.22 mm to 69.73 mm and from 8.30 mm to 63.77 mm. From a posterior view of the left and right sides of the skulls, the values range, respectively, from 15.57 mm to 69.51 mm and from 15.64 mm to 69.44 mm.* Conclusion.* We believe that this study provides additional information about the frequency of elongated SP among the Brazilian population.

## 1. Introduction

**Figure 1:**
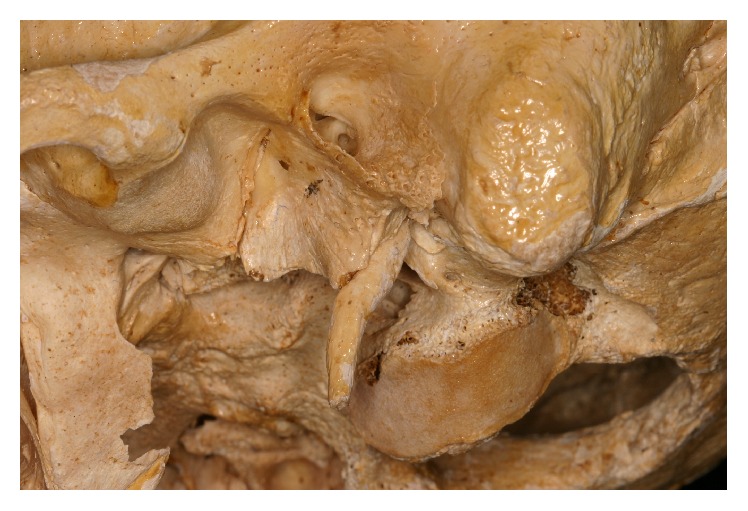
Styloid process.

The styloid process (SP) consists of two narrow and elongated bony projections of the temporal bone. They are located anteriorly to the stylomastoid foramen on the right and left sides of base of the skull [1]. This bone process presents three muscles (stylopharyngeus, stylohyoid, and styloglossus) and two ligaments (stylohyoid and stylomandibular) attached to it [2] (Figure 1).

The length of SP ranges from 15.2 mm to 47.7 mm [3], although several authors have reported that the normal length of this structure is between 20 mm and 30 mm [4–8], with length longer than 30 mm considered elongated. This elongation of the styloid process or calcification of ligaments, which is the cause of the stylohyoid syndrome or Eagle syndrome, was first reported by the otorhinolaryngologist Eagle (1937) [9]. Patients diagnosed with Eagle syndrome may present dysphagia; pain usually focuses on the angle of the mandible and worsens during the rotation of neck or protrusion of the tongue [9, 10]. However, the condition is usually asymptomatic and observed incidentally on a radiographic exam [3, 11]. The relationship between SP and hyoid bone forms the anatomical basis for the glossopharyngeal neurological symptoms associated with elongated styloid process syndrome [12]. Commonly this affects adults bilaterally but may affect only one side of the head [13].

The location of this process is extremely important because it relates to important neurovascular structures. SP is located thereafter in the wall of pharynx and between internal and external carotid arteries and the internal jugular vein. Furthermore, the glossopharyngeal, facial, accessory, hypoglossal, vagus, and other nerves present trajectories that are next to SP. The elongation of this process can cause irritation in various structures next to it [14].

Diagnosis can be made clinically if the elongated styloid process is palpable in the ipsilateral tonsillar fossa. However, the panoramic radiograph requested for other reasons often points to the diagnosis of the syndrome, considering that most patients are asymptomatic [15]. An accurate clinical and radiological evaluation and an experienced professional are important to perform the correct diagnosis. The treatment may be performed by an extraoral or intraoral surgical approach [16].

The aim of the present study was to evaluate measures of the styloid process on Brazilian dry skulls.

## 2. Methods

A total of 109 dry skulls held by the Morphology Department, Institute of Biological Sciences, Federal University of Minas Gerais, were analyzed. Included in the study were specimens that exhibited the two styloid processes without damage and/or fracture signs. An exclusion of 94 dry skulls was necessary, resulting in a sample of 15 skulls.

Two points were determined to standardize measurements, which were obtained using a digital caliper by three observers. The measures were as follows.

**Figure 2:**
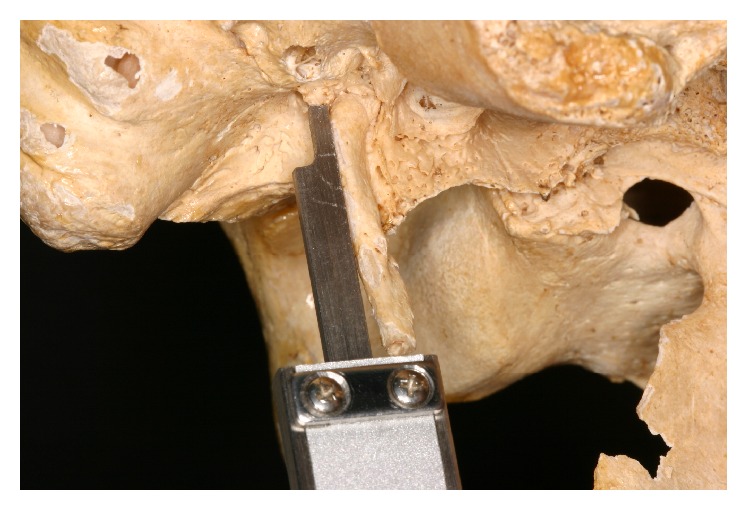
Posterior view.

*(1) Posterior Measure.* This was obtained by measuring the distance from the tip of the styloid process to the flat surface on the side of stylomastoid foramen (Figure 2).

**Figure 3:**
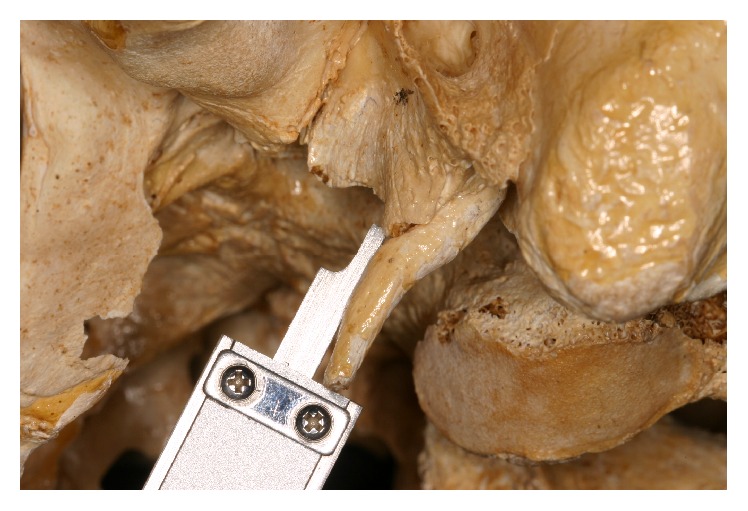
Lateral view.

*(2) Side Measure.* This was obtained by measuring the distance from the tip of the styloid process to its base, on the side of it, where it joins the surface of the anterior wall of the ear canal (Figure 3).

### 2.1. Statistical Analysis

The measures of three observers were synthetized in only one mean measure. Descriptive statistics involved the calculation of central tendency and dispersion measures for each side of the styloid process and for each anatomic position (posterior or lateral). We checked for the normal distribution of each variable, using Shapiro-Wilk tests (*p* < 0.05). We compared the measures for the both sides of the styloid process in each anatomic position, using Wilcoxon tests (*p* < 0.05).

## 3. Results

There was a large variability in the length of left and right sides of the styloid process (in lateral and posterior views). There was no normal distribution for these measures (*p* < 0.001). The central tendency measures of each side in each view are presented on Table 1. There was no difference in the length of styloid process when the sides were compared for each view (*p* > 0.05). The measures obtained by the observers are presented in Table 2.

## 4. Discussion

During embryological development, the SP came from Reichert's cartilage of the second pharyngeal arch [17]. Its length ranges from 15.2 mm to 47.7 mm [3], but other studies have found different dimensions: Jung et al. (2004) [18] suggested that the length of this bone process was longer when it presents more than 45 mm. These variations can occur due to the technique used to measure this length.

There are a variety of ways to determine the dimensions of SP and diagnose Eagle syndrome: panoramic radiographs, X-ray lateral views of the neck, orthopantomograms, and computed tomography. In addition, many studies are based on measurements of dry skulls or cadavers. In some cases the elongated SP can be clinically detected by palpating the tonsillar fossa [19]. Eagle syndrome, or elongated styloid process syndrome, is associated with such symptoms as chronic facial and neck pain, dysphagia, tinnitus, referred pain in the ear, glossopharyngeal neuralgia, orbital pain, and radiating pain in the maxillary regions, which worsen when the head rotates or the tonsillar fossa region is palpated [20].

The cause of elongation of SP is poorly understood, but the most common theories propose congenital elongation of the styloid process, calcification of the stylohyoid ligament by an unknown process, and growth of osseous tissue where the stylohyoid ligament inserts [11, 17].

**Figure 4:**
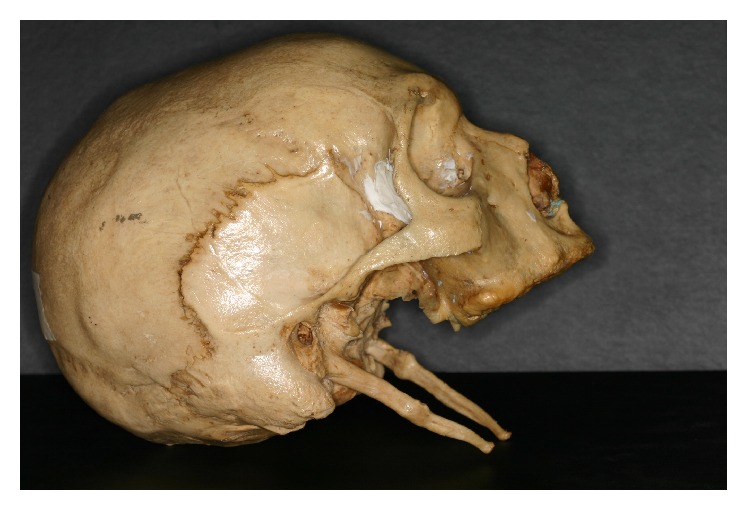
Elongated SP (skull E1 of the study).

Understanding the frequency of elongated SP in Brazil can help clinicians diagnose Eagle syndrome and treat it. In the present study, the length of the SP ranged from 10.22 mm to 69.73 mm and from 8.30 mm to 63.77 mm, based on the lateral view of the left and right styloid, respectively. From the posterior view, the values for the left and right sides of the skulls, respectively, ranged from 15.57 mm to 69.51 mm and from 15.64 mm to 69.44 mm. According to the three observers in this study, from the right to the left view, the mean length of the styloid process was 19.25 mm and 18.90 mm in the lateral view and 24.98 mm and 26.04 mm, in the posterior view. The normal length of SP varies in the literature from 15.2 mm to 47.7 mm, according to Moffat et al. (1977) [1]; measures less than 30 mm, according to Kaufman et al. (1970) [21]; and measures from 20 mm to 30 mm, according to Lindeman (1985) [22]. Considering the normal length of SP as defined by Eagle (i.e., 25 mm to 30 mm) (1937) [9], the presence of one elongated SP was observed in this research among 15 dry skulls with a prevalence rate of 6.6% of the total analyzed (Figure 4). The prevalence of elongated styloid process in the earlier studies was 1% [23], 4% [24], and 8.2% [25]. The 4% prevalence rate in the present study is similar to the rate observed by Eagle. Other Indian studies by Rathva et al. (2013) [26] reported the prevalence of elongated styloid process as up to 2%.

Other authors report that the Eagle syndrome is not only a long stylohyoid process; other factors are necessary for a diagnosis [27].

The pathophysiological mechanisms for the pain associated with Eagle syndrome include

1. compression of the neural elements (i.e., glossopharyngeal nerve, lower branch of the trigeminal nerve, and/or the chorda tympani);
2. fracture of the ossified stylohyoid ligament, followed by inflammatory reaction;
3. impingement on the carotid vessels by the SP, producing irritation of the sympathetic nerves in the arterial sheath;
4. degenerative and inflammatory changes in the tendinous portion of the stylohyoid insertion;
5. irritation of the pharyngeal mucosa by direct compression;
6. stretching and fibrosis involving the fifth, seventh, ninth, and tenth cranial nerves in the posttonsillectomy period [28].

## 5. Conclusion

The study and knowledge of the anatomical variations of the SP in a population may help clinicians from various specialties to diagnose Eagle syndrome. Knowledge of this disorder can prevent the worsening of the painful symptoms related to the elongated SP. We believe that this study provides additional information about the frequency of elongated styloid process in the Brazilian population. Nevertheless, the actual research would be more accurate with a larger number of samples. Another limitation of the present study is the lack of gender-related variation which was not taken into consideration.

## Figures and Tables

**Table 1:** Measurements of the styloid processes, considering the right and left sides of the skulls.

	Left styloid process length (lateral view)	Right styloid process length (lateral view)	Left styloid process length (posterior view)	Right styloid process length (posterior view)
Mean (SD)	19.25 (14.54)	18.90 (13.14)	24.98 (12.94)	26.04 (12.65)
Minimum	10.22	8.30	15.57	15.64
Median	14.87	14.37	23.11	24.01
Maximum	69.73	63.77	69.51	69.44

*p* value^*∗*^	0.865		0.088	

^*∗*^Wilcoxon test.

**Table 2:** Measurements of the styloid processes by observers.

Skull	Measurements in milimeters					
	Examiner 1		Examiner 2		Examiner 3	
	Lateral	Posterior	Lateral	Posterior	Lateral	Posterior
E1 left	**69,80**	**69,39**	**69,47**	**69,54**	**69,93**	**69,60**
E1 right	**63,65**	**69,69**	**63,88**	**69,21**	**63,78**	**69,41**
E2 left	14,41	23,05	14,58	23,18	14,10	23,25
E2 right	14,16	25,46	14,63	25,90	14,31	25,50
E3 left	14,91	23,08	14,80	23,13	14,89	23,11
E3 right	13,25	24,25	13,17	24,24	13,82	24,37
E4 left	10,45	15,71	10,14	15,55	10,07	15,45
E4 right	8,21	15,66	8,55	15,40	8,14	15,85
E5 left	20,50	23,87	20,32	23,91	20,56	23,98
E5 right	17,60	26,68	17,57	26,93	17,64	26,87
E6 left	14,30	20,40	14,35	20,33	14,46	20,51
E6 right	13,62	19,88	13,50	20,16	13,62	19,89
E7 left	11,37	17,65	11,47	17,91	11,32	17,82
E7 right	13,63	18,53	13,73	18,06	13,92	18,04
E8 left	23,15	25,53	22,92	25,46	22,98	25,51
E8 right	24,48	26,54	25,02	26,52	24,49	26,56
E9 left	20,46	29,40	20,71	29,31	20,80	29,12
E9 right	21,53	30,75	21,80	31,00	21,46	30,80
E10 left	16,08	22,09	16,28	22,30	16,31	22,18
E10 right	16,98	21,77	16,71	21,25	16,78	21,40
E11 left	14,44	20,46	14,71	20,38	14,30	20,55
E11 right	13,76	19,80	13,80	20,05	13,95	19,98
E12 left	10,81	16,08	10,69	16,40	10,62	16,26
E12 right	11,55	24,14	11,27	23,96	11,23	23,93
E13 left	19,36	25,31	19,08	25,15	19,44	25,19
E13 right	19,91	25,60	19,98	25,70	19,97	26,07
E14 left	18,78	25,75	18,70	25,86	18,51	25,50
E14 right	19,14	23,35	19,02	23,47	19,26	23,42
E15 left	10,33	16,63	10,29	16,73	10,30	16,70
E15 right	11,76	18,82	11,21	18,43	11,13	18,54
